# Parasite diversity at isolated, disturbed hydrothermal vents

**DOI:** 10.1098/rspb.2023.0877

**Published:** 2023-06-14

**Authors:** Lauren N. Dykman, Carolyn K. Tepolt, Armand M. Kuris, Andrew R. Solow, Lauren S. Mullineaux

**Affiliations:** ^1^ Biology, Woods Hole Oceanographic Institution, MA 02543, USA; ^2^ Massachusetts Institute of Technology, MA 02139, USA; ^3^ Ecology, Evolution, and Marine Biology, University of California, CA 93106, USA

**Keywords:** disturbance, diversity, hydrothermal vents, biogeography, life cycles, parasites

## Abstract

Habitat isolation and disturbance are important regulators of biodiversity, yet it remains unclear how these environmental features drive differences in parasite diversity between ecosystems. We test whether the biological communities in an isolated, frequently disturbed marine ecosystem (deep-sea hydrothermal vents) have reduced parasite richness and relatively fewer parasite species with indirect life cycles (ILCs) compared to ecosystems that are less isolated and less disturbed. We surveyed the parasite fauna of the biological community at the 9°50′N hydrothermal vent field on the East Pacific Rise and compared it to similar datasets from a well-connected and moderately disturbed ecosystem (kelp forest) and an isolated and undisturbed ecosystem (atoll sandflat). Parasite richness within host species did not differ significantly between ecosystems, yet total parasite richness in the vent community was much lower due to the low number of predatory fish species. Contrary to expectation, the proportion of ILC parasite species was not lower at vents due to a high richness of trematodes, while other ILC parasite taxa were scarce (nematodes) or absent (cestodes). These results demonstrate the success of diverse parasite taxa in an extreme environment and reinforce the importance of host diversity and food web complexity in governing parasite diversity.

## 1. Introduction

Habitat disturbance and isolation are important drivers of patterns in biological diversity [1–6]. The immediate consequence of disturbance is usually a reduction or elimination of biomass and abundance, which may correspond to a local loss of species [7]. Disturbance can, however, have a positive net effect on biological diversity over time since it clears space and eliminates competition, allowing different species to establish or become dominant [1,2]. Habitat size and isolation are critical factors in recovery after disturbance [4]. Species are less likely to arrive and establish on smaller, more isolated islands, which can slow recovery rates and result in a lower diversity at equilibrium [5]. Biological traits, such as feeding ecology, symbioses, dispersal and reproduction, help determine when species arrive and establish [6]. While the responses of biological diversity to disturbance and isolation have been tested in many, primarily free-living, functional groups, little is known about the response to disturbance of obligate symbionts such as parasites.

Parasites are increasingly recognized as a dominant component of global biodiversity [8,9] and an important consumer group that can have a keystone influence on ecosystems [10]. They modify the survival, phenology and behaviour of individual hosts [11], alter the demographics and distribution of host populations [12], influence the composition, structure and function of biological communities [13–15], and drive host evolution [16]. Increasing evidence suggests that ecosystems without parasites would function entirely differently [17,18].

Habitat disturbance and isolation can drive parasite diversity directly, by slowing parasite introduction and establishment, or indirectly, by influencing host diversity through the same mechanisms. Parasite richness is tightly coupled to host richness [19], since hosts provide necessary food and habitat, and many parasites specialize on a limited range of host species. The lower host richness often observed on small, isolated islands is theorized to result in shorter, simpler food webs [20] that result in limited niche space for high trophic-level consumers, such as predators and parasites [21,22]. Parasites, like other obligate symbionts, may be more tightly bounded by geographical constraints than are their hosts because the critical steps of dispersal and colonization rely on appropriate hosts being available at the right time. For example, parasites sometimes lag behind their hosts during range expansions [23,24], or are left behind during species introductions [25,26]. In remote, island-like habitats, where species must colonize from afar, parasites may not arrive with initial founder host individuals, or may not become established until hosts reach a threshold population size [27].

Theory suggests island-like habitats may filter certain types of parasites based on their life histories [28]. For example, parasites with indirect (multiple-host) life cycles (ILCs) may be slower to invade or establish in isolated habitats than parasites with direct (one-host) life cycles (DLCs) because they require multiple, sometimes obligate, host species to first establish and reach threshold densities [29]. A decreased diversity of ILC parasites has also been observed after disturbances that alter host diversity and abundance [30], such as the removal of top predators by fishing [31]. Conversely, the diversity of ILC parasites has been shown to increase with host diversity after habitat restoration [32].

Deep-sea hydrothermal vents are island-like, ephemeral habitats, making them a compelling system in which to study the impact of isolation and disturbance on parasite diversity. The geothermal activity that fuels vent communities is patchily distributed, resulting in small habitat areas which support discrete populations connected by dispersal [33]. Venting activity can be dynamic, with relatively short-lived habitat patches compared to other island systems [34]. On fast-spreading plate boundaries such as the East Pacific Rise (EPR), large volcanic disturbances appear to occur on decadal time scales [35] and eradicate animal life over several square kilometres [36,37]. This requires the entire community to reassemble by colonization from surrounding undisturbed sites, a process which may continue for decades [38,39]. The island-like nature of vent habitats poses dispersal and colonization challenges for all vent life, but is likely to be particularly difficult for parasites due to their reliance on hosts. If parasites lag behind hosts in colonization and establishment, certain parasite species may be slow to establish or fail to persist in vent metacommunities where habitat patches are frequently disturbed or far apart.

Features of the biological communities at vents may further limit the colonization, establishment or persistence of parasite species. First, vent communities are characterized by a relatively low richness of free-living species [40]. Free-living species richness is a known driver of parasite richness [19], so a low number of free-living species reduces opportunities for parasite introductions into vent ecosystems with founder host species and constrains the niches into which parasites can radiate once established. Second, vent communities have relatively simple food webs and a low number of vertebrate predator species [41]. For example, vent fields on the northern EPR have only two endemic fish species [42]. Parasite taxa with ILCs have one or more life stages that pass up food chains when one host consumes another. These include helminth parasites such as acanthocephalans (thorny-headed worms), cestodes (tapeworms), nematodes and some trematodes (flatworms). They live as encysted intermediate life stages (cystacanths, metacestodes, larvae and metacercariae, respectively) in invertebrate and vertebrate prey (intermediate host), and mature as sexually reproducing adults in a vertebrate predator (definitive host). The relatively low richness of endemic fauna at vents is expected to limit the number of parasite species with ILCs, particularly interfering with life cycles that pass up food chains through multiple vertebrate hosts.

Consistent with the expectation that features of vent ecosystems may limit parasite diversity, few parasite species have been reported from vents. The most recent review of metazoan vent parasites worldwide [43] includes only seven described species and three unpublished accounts. Since this review, an additional five copepods, five trematodes, one monogenean, one nematode and one isopod have been reported in published literature (see electronic supplementary material, figure S1) [44]. The rate of less than one new parasite species per year is remarkably low compared to the over 700 new free-living species (approx. 42 species per year) described since the discovery of animal life at vents in 1977 [45].

Although isolation, disturbance or simple food webs might limit parasite richness at vents, the scarcity of reported vent parasites may simply be due to low research effort. Relatively few studies have sampled for parasites from host populations in deep benthic ecosystems [46–48], even fewer from a host population at vents or hydrocarbon seeps [49,50], and none from a comprehensive range of host taxa from a single vent community. Without quantitative, community-level data, it remains impossible to test whether parasite richness is truly reduced at vents or to draw any meaningful conclusion as to whether distinct factors drive parasite richness at vents relative to other ecosystems.

We quantitatively sampled the biological community at the 9°50′N hydrothermal vent field on the East Pacific Rise (EPR 9N) to test fundamental theories on parasite richness in a novel, extreme ecosystem. Its location on a fast-spreading plate boundary that experiences large-scale eruptive disturbance every 10–20 years [35] allows us to examine how features of parasite species richness and composition differ in an island-like and disturbed environment. We compare our data to coastal marine benthic ecosystems that differ in their degree of disturbance and isolation, and for which parasitological data were collected using similar methods: kelp forests on the coast of Santa Barbara, California, USA [51] and the lagoon sandflat of Palmyra Atoll in the Line Islands Archipelago [52–56].

We use these data to first test the hypothesis that parasite richness will be lowest in an island-like, disturbed habitat (deep-sea hydrothermal vent), intermediate in an isolated, undisturbed habitat (atoll lagoon sandflat) and highest in a well-connected, moderately disturbed habitat (kelp forest). Second, we ask whether there are relatively more parasite species with DLCs (one host) than ILCs (multiple hosts) at vents due to the additional challenges multi-host parasites must overcome to establish and persist. Finally, we test whether the vertebrate top predators at vents (fish) have a lower richness of intermediate parasite life stages to explore whether simple food webs may limit the trophic pathways that ILC parasites use to complete their life cycles. Although ecosystems with community-level parasite data are currently few and our study is limited to comparing one example of each ecosystem, our inclusion of hydrothermal vent habitats in this study substantially expands the scope and synthesis of parasite investigations across a disturbance spectrum.

## 2. Methods

### (a) Biological collections

Animals were collected from deep-sea hydrothermal vent sites at the EPR 9°50′N vent field (EPR 9N) during two cruises, one in December of 2019 (AT42-21) using HOV *Alvin* and one in March–April of 2021 (RR2102) using ROV *Jason*. In this region, ‘vent sites’ are discrete areas of warm-water outflow. The faunal community within a vent site usually extends approximately 100 m around a central venting orifice [57]. Individual vent sites are separated by tens to hundreds of metres of bare basalt characterized by relatively low faunal biomass and species composition more similar to the ambient seabed than an active vent community [57]. Our samples were collected 13–15 years after a massive seafloor eruption in 2006 paved over vent sites at the EPR 9N vent field [37], after which their biological communities had to completely reestablish.

We collected potential hosts from 11 vent sites including nine in the 9°50′N vent field proper (9.8300, −104.2900, depth 2500 m): Bio-vent, M Vent, Zeta Garden, Teddy Bear, Crab Spa, Tica Vent, Riftia Mound, P Vent, East Wall; and two in the 9°47′N vent field approximately 7 km further south (9.7750, −104.2802, depth 2520 m): V Vent and L Vent (see electronic supplementary material, figure S2). We sampled hosts from a range of thermal zones [58] and substrate types, including basalt and active sulfide chimneys, to capture the range of primary potential host species in the ecosystem and to increase the chance of encountering parasite species that are patchily distributed in host populations. Large tubeworms and mussels were collected into sealed, insulated ‘bioboxes’ using the manipulators of the deep submergence vehicles HOV *Alvin* and ROV *Jason*. Mobile hosts such as fish, crabs and squat lobsters were collected using suction samplers or box-style crayfish traps baited with tubeworm and mussel tissue and left overnight on the seafloor. Small crustaceans, mollusks and polychaetes were gathered opportunistically from geological samples or directly targeted with suction sampling or grabs. During these cruises, we were only able to collect limited numbers of the two endemic fish species at EPR 9N (nine *Thermarces cerberus* and two *Thermichthys hollisi*). We supplemented our data with fish specimens collected from the same sites during prior cruises: two whole *T. cerberus* from 2017 and guts of 22 *T. hollisi* from 2007 [42].

### (b) Dissections

Potential hosts were dissected fresh aboard the ship or frozen at −80°C immediately after arrival at the surface. Dissections followed standard methods for detecting metazoan parasites [14,51,54] (see electronic supplementary material, section S3). Briefly, all tissues of each host were thoroughly examined by squashing between two glass plates and examining under a dissecting scope illuminated by transmitted light. Gut contents were examined separately before squashing. Metazoan parasites were assigned a species or morphogroup name and counted. Expert taxonomists were consulted to assure morphogroups were distinct to a species level, complemented by DNA barcoding of the 18S and 28S genes (see electronic supplementary material, section S3). If there was uncertainty as to whether a symbiont was parasitic, we determined parasitic status based on three criteria: the symbiont must be (i) found living in close association with a potential host and not elsewhere, (ii) have specialized morphology for feeding or attachment and (iii) be embedded within host tissues and have evidence of feeding on host tissues. All dissection data for the EPR 9N vent field fauna are available in BCO-DMO [59].

### (c) Comparison datasets and parasite life cycle assignment

**Figure 1.**
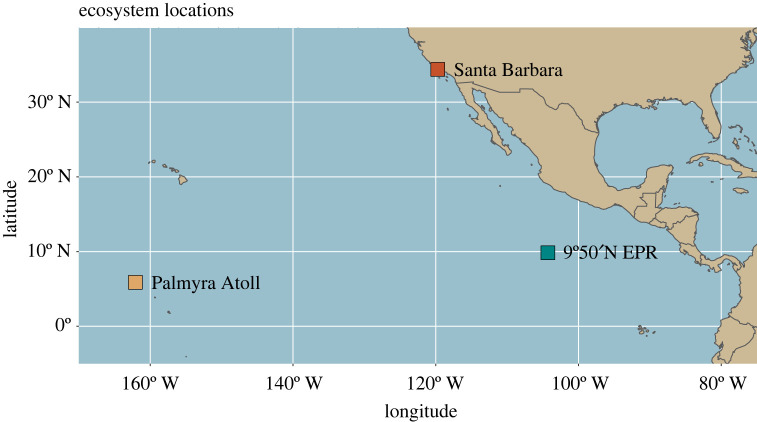
Locations of the three ecosystems: Santa Barbara kelp forests (red); Palmyra Atoll lagoon sandflats (gold); EPR 9N vents (teal). Colour coding for the ecosystems remains consistent throughout the manuscript.

We compared our vent data to similar dissection datasets from two other marine benthic ecosystems: kelp forests on the coast of Santa Barbara, California [51] and the intertidal lagoon sandflats of Palmyra Atoll, Line Islands Archipelago [52–56] (figure 1). Here, we consider benthic species to include those living on the surface of the seabed as well as those living just above, following the convention of the MarLIN BIOTIC database [60]. These ecosystems differ from EPR 9N vents in their combination of disturbance regimes, isolation and the richness of their free-living fauna (table 1). Collectors and dissectors for all three datasets were trained in the same laboratory group and followed similar protocols. In all datasets, specimens were collected haphazardly over several years at a range of collection sites, often targeting the numerically or functionally dominant free-living species in the system. All three studies were designed to be unbiased regarding host species expected to have more or fewer parasites.

**Table 1.** Information on key features of the three ecosystems expected to drive parasite richness, the number of host species and host individuals (in parentheses) included in comparative analyses, and additional geographic and temporal information on specimen collections.

	kelp forest	atoll lagoon sandflat	vent
ecosystem features			
isolation	well connected to kelp forests along the coast and adjacent habitat [51]	extremely isolated; nearest sandflat 376 km away on Kiritimati Atoll [54]	extremely isolated; vent fields spaced tens to hundreds of kilometres apart [33]
disturbance	moderate; storms, wave action and fishing [61]	minimal; no permanent human settlement or fishery, some military development [54]	frequent and intense; seafloor eruptions [35]
food webs	complex; support commercial fisheries, top predators include birds (17 spp.), elasmobranchs (10 spp.) and mammals (4 spp.) [51]	complex; intact with high top predator biomass [62] including birds (6 spp.) and elasmobranchs (3 spp.)	relatively simple; top predators are fish (2 spp.) and crabs (2 spp.) [42,63]
host species analysed			
crustacean	2 (*n* = 25)	5 (*n* = 200)	6 (*n* = 712)
fish	12 (*n* = 195)	23 (*n* = 574)	2 (*n* = 35)
mollusk	12 (*n* = 379)	12 (*n* = 1578)	10 (*n* = 880)
polychaete	2 (*n* = 79)	4 (*n* = 285)	10 (*n* = 507)
total	28 (*n* = 678)	44 (*n* = 2637)	28 (*n* = 2134)
collection information			
collection dates	Oct 2012–July 2017	July 2009–July 2012	Feb 2007–Apr 2021
no. collection days	31	38	25
no. collection sites	8	10	15
geographical range	12 km	4 km	7 km

The three datasets were filtered following a consistent protocol to facilitate comparison (see electronic supplementary material, section S3). Only host species with a sample size of ten or more individuals were included in analyses. We focused on four host taxonomic groups: crustaceans, fishes, mollusks and polychaetes. This study includes metazoan parasites, omitting microbial pathologies and symbionts that are not parasitic. We adopt the ‘consumer strategy’ definition of a parasite as an organism that feeds on only one individual resource at a single life stage [64]. The metazoan parasite groups included in analysis were Acanthocephala, Cestoda, Copepoda, Isopoda, Monogenea, Nematoda, Rhizocephala and Trematoda. Each parasite species or morphogroup retained for analysis was categorized as having a DLC (one host) or ILC (multiple hosts) based on observation (i.e. matching life stages with genetic barcoding) or published literature (see electronic supplementary material, section S3). Life cycle assignments and citations are available in BCO-DMO [65]. Scripts for data subsetting, merging and analysis are available in Zenodo [66].

### (d) Comparing parasite richness at vents to other marine ecosystems

We tested whether two components of parasite species richness are low at deep-sea hydrothermal vents compared to other ecosystems. The first component, parasite richness within host species, examined whether a free-living species at vents will, on average, host fewer parasite species than does a free-living species in the other two ecosystems. Here, we apply an *ad hoc* approach to test the null hypothesis *H_0_* that parasite richness within host groups is the same in the three ecosystems against the alternative hypothesis *H_1_* that parasite richness within host groups follows the ordering kelp forest > atoll sandflat > vent, suggested by biogeography theory. To account for the dependence of observed parasite richness on the number of host individuals examined, parasite richness within host species was estimated with the Chao2 species estimator using the *chao2* function in the R package ‘fossil’ [67], which is the most effective estimator of parasite community richness [68]. We analysed this variable using a nonparametric rank analysis-of-covariance (ANCOVA) model [69] with host group and ecosystem as fixed factors and the rank of average host species length as a covariate (see electronic supplementary material, section S4). This covariate is included to account for the dependence of observed parasite richness on the size of the host individual [70] (see electronic supplementary material, figure S4). Significance was assessed by randomizing the assignment of the species within host groups to ecosystems. All analyses were performed in R Version 4.2.1 [71].

Second, we analysed the relationship between parasite richness and host richness in the three ecosystems using traditional species accumulation curves. The points on these curves represent estimates of mean parasite richness in fixed numbers of host species. Curves were generated using the equations from Solow & Smith [72] (see electronic supplementary material, section S5) with the number of host species as the sampling unit. Separate curves were produced for fish and invertebrate hosts. When analysing parasite richness across host species, one factor that could lead to overestimates of richness is erroneously counting different life stages in ILCs as separate species. Some life stages in the three datasets were not identified to species and were assigned separate morphogroup IDs from the adults. Separating fish and invertebrates for analysis avoided this source of error for ILC species that pass from invertebrates to fish. To account for parasite species that pass through multiple fish species or multiple invertebrate species, we omitted life stages that were potentially redundant with other life stages within fish and invertebrate hosts from species accumulation curves.

### (e) Comparing the proportion of parasite species with direct and indirect life cycles

We tested the null hypothesis that the proportions of parasite species with ILCs are the same across ecosystems using a standard χ^2^ test applied to contingency tables. As with the richness analyses that look across host species, we treated fish and invertebrate host species separately and omitted potentially redundant life stages. This subsetting removed several ILC larval morphogroups from the kelp forest data but did not qualitatively change results. We also generated species accumulation curves for each parasite taxonomic group to examine which parasite taxa drove differences in richness in the two life cycle categories. We focused on fish hosts for this analysis since fish hosted the greatest number of parasite species from the widest range of taxa.

### (f) Evidence of trophic relationships from parasite life stages

We tested the null hypothesis that the proportions of parasite morphogroups in different life stage categories are the same across ecosystems using a standard χ^2^ test applied to contingency tables. As before, we analysed fish and invertebrate hosts separately. We hypothesized that we would find relatively few parasite species in intermediate stages (acanthocephalan cystacanths, larval nematodes, cestode metacestodes and trematode metacercariae) and relatively more in the adult stage in vent fishes, since the short food chains and low richness of vertebrate predators (only two fish species) at EPR 9N vents may preclude parasite species that use fish as intermediate hosts. Since this analysis focuses on life stages rather than total species richness, all intermediate stage morphotypes were included in this analysis.

## 3. Results

### (a) Summary of East Pacific Rise 9N vent fauna surveys

**Figure 2.**
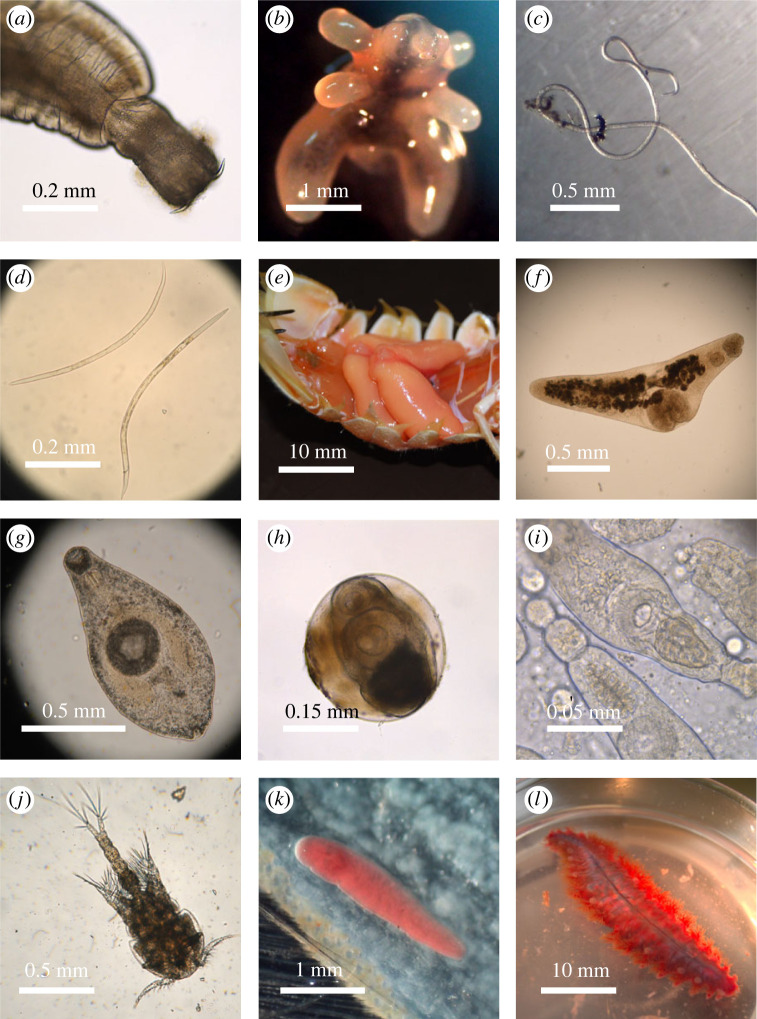
Selected photographs of parasite and other symbiont species encountered in dissections of EPR 9N fauna: (*a*) Acanthocephalan from the gut of the fish *Thermarces cerberus*; (*b*) copepod (family Chondracanthidae) from the mouth of *T. cerberus*; (*c*) nematode (genus *Ascarophis*) from the gut of *T. cerberus*; (*d*) larval nematodes from the amphipod *Ventiella sulfuris*; (*e*) rhizocephalan (genus *Paratriangulus*) in the abdominal muscle of the squat lobster *Munidopsis cf. exuta*; (*f*) adult trematode (family Opecoelidae) from the gut of *T. cerberus*; (*g*) adult trematode (family Opecoelidae) from the gall bladder of *T. cerberus*; (*h*) trematode metacercaria from the abdominal muscle of the shrimp *Alvinocaris lusca*; (*i*) trematode sporocyst from the gonad of the limpet *Eulepetopsis vitrea*; (*j*) commensal or micropredator copepod from the gills of *B. thermophilus*; (*k*) nemertean egg predator, potentially of the genus *Ovicides*, from the claws, legs and gonopods of the crabs *Bythograea thermydron* and *Cyanagraea praedator*; (*l*) scaleworm *Branchipolynoe symmytilida* commensal with the mussel *Bathymodiolus thermophilus*. Note that the scalebars are different in each panel. All scalebars are in millimetres.

We dissected 2,215 potential host individuals of 51 species from the EPR 9N vent field (see electronic supplementary material, table S1). These included 10 crustacean, 2 fish, 14 mollusk and 25 polychaete species. Dissections revealed 12 adult metazoan parasite morphogroups and nine larval morphogroups in five of the major marine parasite taxa included in this study (figure 2). These were two adult acanthocephalans, one adult copepod, one adult nematode, one larval nematode, one adult rhizocephalan, seven adult trematodes, seven trematode metacercariae and one trematode sporocyst. Dissections also revealed non-parasitic symbionts: several copepod species that may be commensals or micropredators, nemertean egg predators and the commensal scaleworm *Branchipolynoe symmytilida*. All dissection data for EPR 9N vent field fauna are available in BCO-DMO [59].

### (b) Comparing parasite richness between marine ecosystems

**Figure 3.**
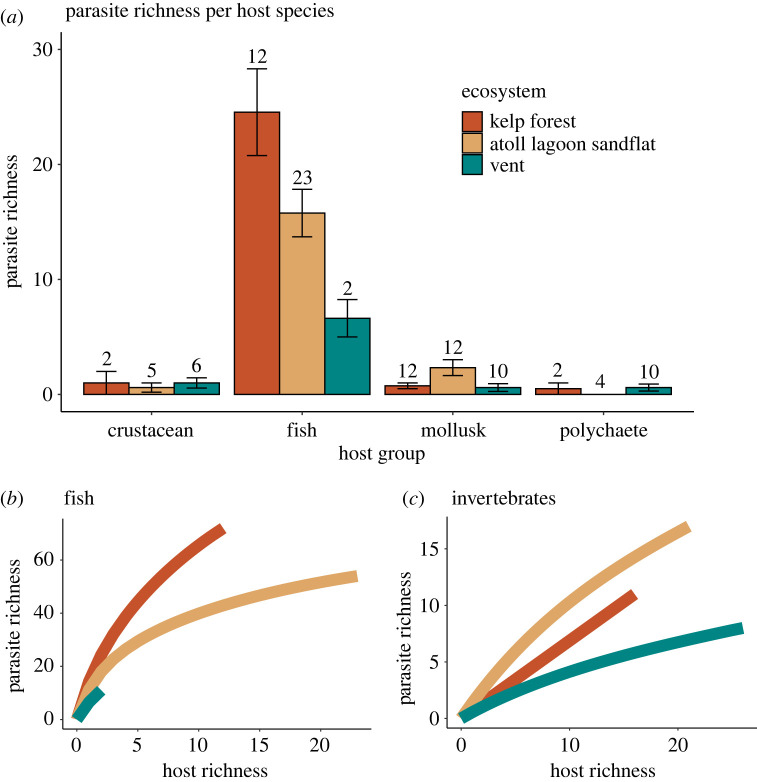
(*a*) Mean (±s.e.) parasite richness estimated by Chao2 within host species in each ecosystem and host group. The number above the error bar indicates the number of host species included in the ecosystem and host group. Parasite species accumulation curves as a function of the number of host species sampled for (*b*) fish hosts and (*c*) invertebrate hosts.

Parasite richness within host species broadly followed the hypothesized order, being highest in kelp forests, intermediate at the atoll lagoon sandflats, and lowest at vents, but these differences were not significant (2-way ANCOVA with ordered alternative, *R*^2^ = 0.67, *p* = 0.39) (figure 3*a*). The relationship between parasite richness and host richness for fish hosts followed a similar trajectory in the three ecosystems (initial slope of the line), but the low number of vent fish species constrained the total number of parasite species in the vent community (endpoint of line) (figure 3*b*). The relationship between parasite richness and host richness for invertebrate hosts was lowest at vents and greatest at the atoll lagoon sandflat (figure 3*c*).

### (c) Comparing the richness of parasites with direct and indirect life cycles

**Figure 4.**
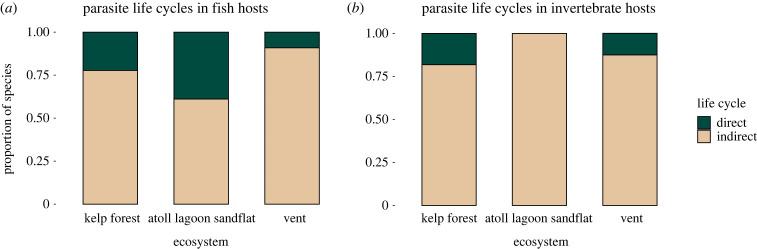
The relative number of parasite species with DLCs and ILCs in the three ecosystems in (*a*) fish hosts and (*b*) invertebrate hosts. In this figure, fish hosts include those used as intermediate and definitive hosts.

Most of the parasite species in each of the three ecosystems had ILCs. The relative number of parasite species with DLCs and ILCs was significantly different between the ecosystems for fish hosts (*χ*^2^ (d.f. = 2) = 6.4, *p* = 0.042) (figure 4*a*) but not for invertebrate hosts (*χ*^2^ (d.f. = 2) = 3.1, *p* = 0.21) (figure 4*b*). The proportion of parasite species with ILCs was greater in vent fish than expected under the null model and was more similar to kelp forests than to the atoll lagoon sandflat.

**Figure 5.**
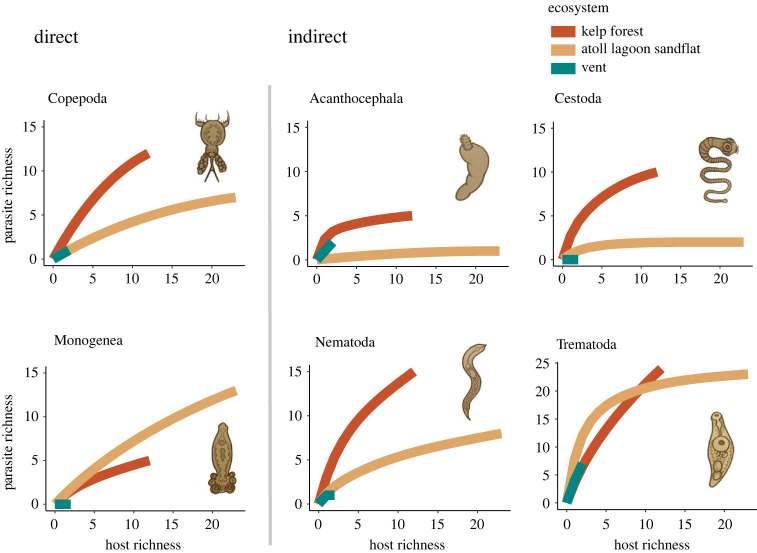
Species accumulation curves for the six parasite taxa found in fish as a function of host richness. Parasite taxa are grouped by whether their species generally have DLCs or ILCs.

Differences between ecosystems in the proportion of DLC to ILC parasite species were driven by a few parasite taxa, and patterns were not consistent across parasite taxa within the same life cycle category (figure 5). Among DLC parasites, copepod richness at vents was similar to the atoll lagoon sandflat while monogeneans were absent at vents. Among ILC taxa, nematodes were low in richness and cestodes were absent at vents, while acanthocephalan and trematode richness was comparable between vents and the coastal ecosystems.

### (d) Composition of parasite taxa and life stages in fish hosts

**Figure 6.**
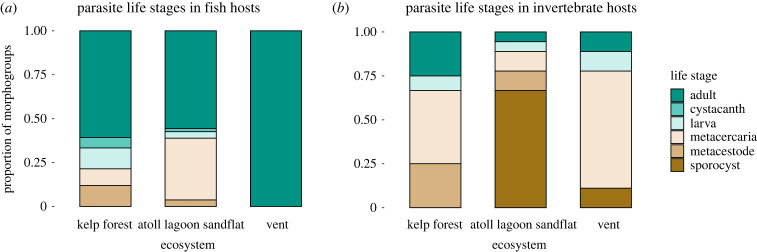
The relative number of morphogroups of different parasite life stages in the three ecosystems in (*a*) fish hosts and (*b*) invertebrate hosts.

The relative number of parasite morphogroups in different life stages was significantly different among the ecosystems for both fish (*χ*^2^ (d.f. = 8) = 26.3, *p* = 0.00093) (figure 6*a*) and invertebrate hosts (*χ*^2^ (d.f. = 8) = 22.2, *p* = 0.0045) (figure 6*b*). Vent fish hosted only adult parasites and lacked the acanthocephalan cystacanths, larval nematodes, trematode metacercariae and cestode metacestodes that were common in fish from other ecosystems. Among invertebrate hosts, vents lacked metacestodes yet had a high richness of metacercarial morphogroups.

## 4. Discussion

### (a) Challenges of recruitment in an island-like, disturbed habitat

The isolation of vent habitats is expected to challenge the recruitment and persistence of parasite species. Differences in parasite richness in free-living species between the three ecosystems were not statistically significant despite following the hypothesized order suggested by island biogeography theory (figure 3*a*). This demonstrates the ubiquity of parasites in biological communities, even in extreme settings. Of the four host groups examined, parasite richness in fish hosts most closely followed the expectation based on habitat isolation (figure 3*a*). Fish probably provide the most integrated sampling of regional parasite richness because they are highly mobile and acquire many of their parasites through feeding, thus accumulating parasites over space and time. Santa Barbara kelp forest fish species likely had the highest parasite richness because their habitat is connected to kelp forests along the coast and interacts with adjacent rocky intertidal, pelagic and estuarine areas [51]. Although fish species in Palmyra sandflats move between some adjacent habitats (coral reef and pelagic), Palmyra Atoll is comparable to vents in its small area (approx. 4 km^2^) and extreme isolation from the nearest sandflat, which is 375 km away on Kiritimati Atoll [54] (table 1).

Kelp forests and atoll lagoon sandflats likely facilitate greater parasite connectivity due to their greater taxonomic richness of highly mobile definitive hosts. The kelp forest food web includes avian (17 spp.) and mammalian (4 spp.) definitive hosts, while the atoll sandflat supports pelagic fishes (approx. 5 spp.) and birds (6 spp.) that can act as long-distance dispersal vectors between islands. The finding that parasite richness in vent hosts was not statistically different compared to hosts in less isolated and less disturbed systems suggests that vent fishes may be effective dispersers and offer more connectivity between vent fields than has been appreciated. The extent to which vent fishes move among vent fields is unknown; however, all available evidence suggests that they are closely associated with vent ecosystems. The feeding spheres of the two fish species at EPR 9N, *T. cerberus* and *T. hollisi*, have been inferred from otolith chemistry [42] and gut contents [73] and are unlikely to extend into adjacent habitats or other depths in the water column [63].

Extreme, frequent disturbance is another factor expected to reduce parasite richness at EPR 9N vents. Patterns of parasite richness in invertebrate hosts (figure 3*c*) correlated with the relative levels of disturbance in the three ecosystems, with Palmyra being least disturbed and vents being the most. Differences in parasite richness between fish and invertebrate hosts could result from their distinct modes of infection: fish are often infected by feeding on other hosts, whereas invertebrates are infected by encountering dispersive parasite stages. Parasites may take longer to colonize and spread in the invertebrate community after disturbance, since transmission among invertebrates is more a function of relative host densities than of host feeding rates. Since our samples of the vent community were taken 13–15 years after the 2006 eruption, when the community was already in advanced stages of recovery [38,39], time since the disturbance does not entirely explain the reduced parasite richness in vent invertebrates. Local hydrographical conditions may also influence the probability of parasite larvae encountering a host; vents and kelp forests are open systems with significant water flow, while the atoll lagoon is a more enclosed system with greater entrainment.

### (b) Successful life histories in island-like, disturbed habitats

Due to challenges of recruitment in island-like, ephemeral habitats, we expected to find relatively more parasite species with DLCs (one host) than ILCs (multiple hosts) at vents compared to other ecosystems [29]. Instead, we found more parasite species with ILCs than expected at vents (figure 4). This result was driven by the high richness of trematodes and the absence of monogeneans (figure 5) and does not support the theoretical expectation that island-like habitats filter parasite species based on the number of hosts they use in their life cycles. Rather, our results indicate that the establishment and persistence of parasites on islands is determined by the specific ecological requirements of parasite taxa, including opportunities for introduction and life cycle completion within the available host species [74].

Some aspects of ILCs may be advantageous in disturbed habitats, allowing certain taxa to persist. In general, ILCs allow parasites to maximize different fitness costs at different stages, with higher transmission rates in small intermediate hosts (which are often more abundant) and greater growth and reproduction in definitive hosts (which are often large and long-lived) [75]. Trematodes may be particularly successful in disturbed environments because they have an asexual cloning stage, which allows for multiplication in small intermediate hosts, while also using fish, a highly mobile host group, for long-distance dispersal. Bottom-up nutrient enrichment in estuarine ecosystems is associated with high trematode densities, likely because it increases the abundance of gastropod intermediate hosts [76]. Trematode species richness and biomass are also high in these estuarine ecosystems [14,77]. This same mechanism may enhance trematode transmission at vents, where venting activity provides a rich bottom-up source of energy with an abundance of gastropods as first intermediate hosts.

DLC parasites were less species rich at vents than expected, as we encountered only one parasitic copepod and one rhizocephalan species. Monogeneans were conspicuously missing at vents despite being speciose in the other ecosystems and known from fish hosts as deep as 5000 m [46]. Their absence may be explained by ineffective larval dispersal, low host densities, high host specificity or sensitivity to the chemical environment. The low richness of DLC parasites at vents could indicate that reliance on a single, often highly specific, host species may be a relatively inefficient strategy in isolated disturbed environments. Finally, parasite species in the absent DLC taxa—isopods and monogeneans—may be present at vents at very low prevalence and might be discovered with greater sampling effort.

### (c) The effect of simple food webs and low richness of top predators

The relatively low host richness and short food chains at vents [40,41] are expected to restrict parasite richness overall [19], but especially limit the potential pathways for ILC parasites to complete their life cycles. Our data reveal that vent fish lack the encysted intermediate parasite stages of acanthocephalans, cestodes, nematodes and trematodes that are common in marine fish in other ecosystems (figure 6*a*). These differences cannot be explained by the deep-sea setting alone, since metacestodes and larval nematodes have been found in fish from 1000 to 5000 m depth [46,78]. The absence of intermediate parasite life stages in vent fish is likely due to the ecosystem having only two vertebrate predatory species. Kelp forests and the atoll lagoon sandflat have many more fish species (56 spp. and 38 spp., respectively) and potential trophic links than do vent ecosystems, plus they include other vertebrate taxa—sharks, birds and marine mammals—as final hosts. For a specific example, most marine cestodes use sharks as definitive hosts. The absence of cestodes at vents may result from sharks avoiding vent habitat or a scarcity of deep-sea sharks in the EPR region. Taken together, richness trends within the limited number of fish hosts (figure 3*b*), and the absence of intermediate parasite life stages in vent fishes (figure 6*a*), suggest that total parasite richness in the community is largely constrained by the available number of host species and trophic interactions. Low predator diversity may be a direct result of the small habitat area [21,22] or the challenges of vertebrate species adapting to tolerate a harsh chemical environment.

## 5. Conclusion

Broadening comparative studies to extreme environments is useful for testing the generality and exceptions of ecological patterns and offers insights into causes for exceptions. Our study reveals that parasite richness in vent hosts is comparable to two coastal marine ecosystems, despite vent habitat being more isolated and more disturbed. This indicates that the previously low encounter rate of vent parasites was largely due to lack of sampling effort, and provides additional evidence of the ubiquity of parasites, even in extreme environments. Contrary to expectation, parasites with ILCs were relatively speciose at vents due to the success of trematodes, whose life strategy may allow for effective dispersal and rapid establishment. The low host richness at vents and the low number of trophic pathways available for parasites to complete their life cycles were found to be important regulating factors for parasite richness and may explain the scarcity or absence of some ILC parasite taxa at vents. Since our study compares three very different ecosystems and is limited by only having one example of each ecosystem, the generality of these findings should be tested in other settings that lie along a spectrum of disturbance and isolation. Finally, EPR 9N is disturbed at a high frequency that is not typical for most vent fields, so additional insight into processes will be gained by investigating more vent fields with different disturbance frequencies and assemblages of potential host species.

## Data Availability

Dissection data from EPR 9N hydrothermal vent fauna are available in the publicly accessible BCO-DMO database (https://www.bco-dmo.org/) as doi:10.26008/1912/bco-dmo.879118.2 [59]. Dissection data from kelp forests are available in Morton *et al*. [51]. Dissection data from Palmyra Atoll Sandflats are part of an in-prep data paper that is not yet published. The list of Palmyra parasite species included in this analysis is published in Vidal-Martínez *et al*. [52], Vidal-Martínez *et al*. [53], McLaughlin [54], González-Solís *et al*. [55] and Soler-Jiménez *et al*. [56]. Life cycle assignments and citations for all parasite species included in analyses are available in BCO-DMO as doi:10.26008/1912/bco-dmo.879253.2 [65]. Data on the vent parasite species reported in literature prior to this study are available in BCO-DMO as doi:10.26008/1912/bco-dmo.879266.1 [44]. Scripts used for this analysis are available in Zenodo at https://doi.org/10.5281/zenodo.7938924 [66]. The data are provided in the electronic supplementary material [79].
